# Benevolent Ageism: Exploring Its Boundary Conditions, Generalizability, and Correlates

**DOI:** 10.1093/geroni/igaa057.1882

**Published:** 2020-12-16

**Authors:** Toni Bisconti, Jennifer Sublett, Alison Chasteen

## Abstract

Ageism is one of the few prejudices that is still socially condoned (Nelson, 2016). Given the aging population and the impact of internalizing ageist thoughts, this construct needs to be at the forefront for scientific examination. The long-term effects of ageism, particularly negative self-perceptions, lead to negative health and cognitive outcomes (Chasteen et al., 2015; Levy et al., 2002). One of the intricate components of ageism, however, is that it is often “benevolent”. Cuddy and colleagues developed the Stereotype Content Model (SCM) to describe how individuals are categorized based on varying degrees of warmth and competence. Unlike many devalued members of society who are viewed as low on both, older adults are viewed as having high warmth and low competence, leading to more overaccommodative treatment. The goal of the present symposium is to overview the ways in which researchers have dissected this more nuanced type of ageism. Specifically, two of the presenters will cover some of the boundary conditions of understanding age-based stereotypes and their malleability, examining them across ages and across genders. Additionally, one of our presenters will overview the validation of the Ambivalent Ageism Scale on a Chinese sample, lending support to its generalizability. Finally, our last presenter will overview the relationship between benevolent ageism and self-compassion to predict metamemory, given the pervasive stereotype that older adults suffer from severe cognitive decline. Themes and implications of these presentations will be discussed.

